# Hemophilia Patient Experience in a Physical Therapy-Guided Health Education Intervention: A Mixed-Method Design

**DOI:** 10.3390/healthcare9121728

**Published:** 2021-12-14

**Authors:** Alicia López-Casaus, Carolina Jiménez-Sánchez, Paula Cordova-Alegre, Fani Alfaro-Gervon, Laura Esteban-Repiso, Raquel Lafuente-Ureta

**Affiliations:** Department of Physical Therapy, Faculty of Health Sciences, Universidad San Jorge, Autovía A-23 Zaragoza-Huesca Km. 299, Villanueva de Gállego, 50830 Zaragoza, Spain; alicialopezcasaus11@gmail.com (A.L.-C.); pcordova@usj.es (P.C.-A.); falfaro@usj.es (F.A.-G.); liesteban@usj.es (L.E.-R.); rlafuente@usj.es (R.L.-U.)

**Keywords:** hemophilia, online health education, physical therapy, mixed methods study

## Abstract

People with hemophilia usually have negative joint consequences due to their illness. Evidence suggests that exercise and therapeutic education bring some benefits. An important factor that affects health interventions was the experience and degree of satisfaction. Thus, it is relevant to analyze qualitative and quantitative data to obtain a complete view of the patient’s experience. As a result, a concurrent nested mixed method with quantitative predominance study design was carried out. Nine people with hemophilia of Hemoaralar with a homogeneous environment participated in this study. The items evaluated were the level of satisfaction through the GCPC-UN-ESU survey and the experience with healthcare interventions through a focus group. A high level of satisfaction was obtained, but some divergences between quantitative and qualitative data were found. Further research about physical therapy and this type of intervention in people with hemophilia should be considered to better address the impact of living with the disease.

## 1. Introduction

Hemophilia is characterized by the congenital deficiency of blood clotting factors, as the coagulation factor is not synthesized by the body. It is an X-linked recessive non-dominant hereditary pathology. Depending on the missing factor, there are two types of hemophilia: Hemophilia A, corresponding to factor VIII deficiency, and hemophilia B, corresponding to factor IX deficiency (called Christmas disease). Within this classification, it is subdivided according to the plasma concentration, with three categories of hemophilia: mild (5–40%), moderate (1–5%) and severe (<1%) [1,2]. It is estimated that 1/10,000 births suffer from hemophilia, so it is therefore considered a rare disease [3].

The main signs and symptoms are joint-connected, such as chronic pain, atrophy of the periarticular muscles, limitations in the range of motion, deformity, and alterations in gait and proprioception [4,5]. Moreover, with a lower incidence, there are muscle bleeds (hematomas) accompanied by signs such as inflammation, and flexion contracture, leading to alterations in functionality and pain [6].

Hemorrhages are the most common clinical manifestations of hemophilia. Of these, 90% occur in the intra-articular space due to direct or indirect trauma and are known as hemarthroses; they appear mainly in the elbows, knees, and ankles. Bleeding sustained over time causes a higher risk of irreversible degeneration (hemophilic arthropathy) [2,6]. The pathophysiological process causes irreversible damage, as it is a vicious circle which begins with synovial hypertrophy, triggers fragile neovascularization, predisposes to new bleeds that overflow synovial cleanliness, accumulates hemosiderin, and hinders blood resorption. This usually occurs in severe hemophilia [4,5].

There are non-pharmacological therapies that provide numerous benefits to these patients, such as physical therapy, exercise, and lifestyle changes [1,4,6,7]. The main objectives of physical therapy treatment are to prevent new bleeds, restore functionality, improve quality of life, and avoid complications [8,9].

After a bleed, the method “Protection, Optimal load, Ice, Compression, Elevation” (POLICE) is recommended first followed by the progressive reestablishment of muscle strength, starting with isometric exercises, concentric isotonic exercises, eccentric isotonic exercises, open kinetic chain and finally closed kinetic chain [8,9,10,11]. Manual therapy and exercise can be complemented with NMES, TENS, ultrasound, hydrotherapy, and gait reeducation, in addition to the use of therapeutic education to improve the quality of life and the perception of pain in these patients [12,13,14]. Moreover, chronic pain appears in 35–50% of people with hemophilia, with 40% of these patients indicating that health professionals do not treat their pain well, requiring a better approach from a biopsychosocial sphere [12,13].

However, most studies use quantitative measures of physical aspects and do not take into account the perspective of the patient. For this reason, it has been considered necessary for people with hemophilia to describe, both qualitatively and quantitatively, their experience with the applied treatment. For them to be able to do this, it is necessary to provide them with prior knowledge through health education as a tool to manage their disease. Thanks to that, this study will gain another perspective of the patient, which will be relevant for its approach [12,13,15].

The purpose of this study was to determine the degree of satisfaction, describe the experience, and understand the priorities of people with hemophilia in a therapeutic education briefing. Moreover, the study aimed to analyze the perception of patients and family members in healthcare settings.

## 2. Materials and Methods

### 2.1. Study Design

A concurrent nested mixed method with quantitative predominance study design was chosen [16]. It was conducted in accordance with The Declaration of Helsinki and following the “Mixed Method Article Report Standards” (MMARS) [17].

A quantitative approach guided the data collection, while qualitative data were inserted in the quantitative approach. Both data were compared to obtain a more general overview of the satisfaction obtained by the participants with the healthcare provided [18].

This study was approved by the Ethics Committee of the Universidad San Jorge (N°TF07-20/21).

### 2.2. Participants

Patients and family members from the Hemophilia Organization from Aragon and La Rioja (Hemoaralar) in Zaragoza (Spain) were invited to take part in the study, as a representative and homogeneous sample. Inclusion criteria were: aged 16 or over, being a family member or patient with a Hemophilia diagnosis [19], access to either a smartphone or other wireless device, attending the briefing, signing the informed consent and being a membership of Hemoaralar. Exclusion criteria were: not being able to understand or participate in the focus group and not completing the questionnaires.

### 2.3. Sample Recruitment

A purposive recruitment method was used to collect the sample. Ten days before the intervention, participants were recruited by the social worker of the organization by a phone call and using a noticeboard on the social networks of the organization. Participants were informed through the Participant Information Document, which they read and signed before the briefing.

### 2.4. Intervention

The intervention consisted of an online briefing about physical therapy health education in Hemophilia, which took place on the 18th of March 2021 through Microsoft Teams. It lasted for 90 min, including the speech and a final discussion time with the participants, using questions and answers.

The content of the speech was divided into 4 modules: (1) introduction to the most common musculoskeletal pathology in people with hemophilia [6,11]; (2) objectives and advantages of physical therapy in hemophilia [10,20]; (3) evidence-based physical therapy treatments in hemophilia [21,22]; and (4) therapeutic exercise for people with hemophilia [23].

The contents of the briefing were orally exposed by 1 physical therapist and 1 student in the last academic course, with an audiovisual presentation supported by Canva version 2.93.0, which included text, images, and videos to encourage the attention of the participants. Five days after the briefing, participants joined a focus group with study staff. All of the participants were sent 2 consultative files by mail after the briefing: a document with guided exercises and the presentation of the briefing.

### 2.5. Data Collection and Analysis

#### 2.5.1. Quantitative Data

Quantitative data were collected immediately after the briefing through Microsoft Forms questionnaires. Participants were identified by a number to ensure anonymization. To understand participants’ demographic and clinical characteristics, the following data were collected: type of participant (patient/family member), age, gender, type of hemophilia, and severity of the disease.

To evaluate the level of satisfaction with the briefing, the Survey on Satisfaction with Care of Patients with Chronic Disease and their Family Caretakers (GCPC-UN-ESU) was used. This scale is validated in Spanish for the investigation of satisfaction with healthcare in patients with chronic non-communicable diseases (NCD) [24]. It contains 19 items and 4 dimensions: level of satisfaction with care, with the conditions of the service, health education, and the level of loyalty. The survey evaluates the perception of satisfaction with healthcare by people with NCD, and it can be self-completed. It consists of a Likert scale, where 1 means lack of satisfaction and 5 means exceptionally satisfied.

Quantitative data analyses were conducted using IBM SPSS statistics for Windows, Version 25.0 (IBM Corp, Armonk, NY, USA). A descriptive analysis was performed, calculating the frequency and percentage of each variable to assess the scores obtained in the satisfaction scale.

#### 2.5.2. Qualitative Data

Qualitative data were collected in a focus group with the participants. The goal was to explore the experience of the patients and family members with the intervention and their previous experience with healthcare. It took place 5 days after the intervention through Microsoft Teams. Prior to the interview, participants were informed of the development of the focus group and were asked for their approval to record the content of the discussion. Two researchers performed a previous researcher positioning process or “bracketing”, with the aim of ensuring that their previous knowledge and experiences, beliefs and motivation for the research did not influence the data collection and analysis [25]. These members of the study team, trained in qualitative research and without previous relations with the participants, conducted the interview and took field notes. The narrations were textually transcribed into a Microsoft Word document to be analyzed.

Nine opened semi-structured questions were proposed (Table 1) based on the items of the quantitative satisfaction survey, in order to better understand and compare the results in this mixed-method study. The questions were designed to inquire all the relevant information of the experience of the participants. The focus group lasted for one hour, until data saturation when participants stopped providing new information.

A qualitative descriptive approach [26] was used for qualitative data analysis, using textual transcriptions of the participant narrations to justify themes and subthemes. The process of data analysis followed the steps from the thematic analysis [27,28]. Finally, the data were organized according to the dimensions and items of the quantitative survey.

Data triangulation was performed to improve the quality and trustworthiness of the data. Two authors met to compare and discuss codes, subthemes, and themes, refining and redefining codes to resolve disagreements. Then, findings were sent to the rest of the study staff to be compared and discussed to reach a consensus among the researchers [28].

#### 2.5.3. Mixed-Method Data Analysis

Once quantitative and qualitative data were analyzed, they were compared to identify convergences or divergences between the two types of data, therefore validating the findings. This analysis (joint display) allows the results obtained to be confirmed or explained with both sources of data collection [18].

## 3. Results

The participants were members of Hemoaralar. Table 2 shows the sociodemographic data of the sample. Ten participants attended the briefing, but one of them did not take part in the data collection. The sample was finally comprised of 9 participants who completed the survey. Seven participants took part in the focus group, but the rest were not able to participate due to personal issues.

All of the participants were male, aged between 25 and 69 years old; most of them were people with hemophilia (77.8%), predominantly severe hemophilia A, which reveals heterogeneity of the sample. It is noted that one of the participants did not complete the severity data.

### 3.1. Quantitative Results

Table 3 shows the quantitative data from the Survey on Satisfaction with Care of Patients with Chronic Disease and their Family Caretakers (GCPC-UN-ESU), in reference to satisfaction level of the participants.

Most participants had a high level of satisfaction. In the section of kindness of the staff, 1 participant revealed a low level of satisfaction. Furthermore, 1 participant showed a medium level of satisfaction when answering about preparation of the staff, usefulness of the care provided, institutional availability, provisions for the activities, and effectiveness of the administrative conditions. In the section concerning the benefits of the educational activity, 3 participants showed a medium level of satisfaction.

### 3.2. Qualitative Results

After the qualitative analysis, four main themes emerged reflecting the experience of the participants in the educative intervention and their previous experiences. Table 4 details all of the themes and subthemes that emerged from the focus group.

#### 3.2.1. Experience with Health Care

Participants narrated their experience in the healthcare setting, speaking about the care provided by and treatment received from different professionals.

**Treatment by health professionals**

Participants narrated how they have been treated, claiming a closer and more personal treatment.

“…apart from the usual: there are sick individuals, not diseases to be treated”.

(P.2 (person with hemophilia) 69 years-old)

**Lack of trust in the health professionals**

The participants spoke about the lack of confidence that the professional had sometimes generated in them.

Many times, you go to the general doctor, and you nearly think: “I should go directly to Hospital Miguel Servet to the Hemophilia Unit…because this doesn’t work”.

(P.6 (parent) 49 years-old)

**Perception of the training of professionals**

Some patients explained that they felt a lack of interest and knowledge from the health professionals. They felt that it is necessary for them to know what is recommended and discouraged, and considered it important that they are informed, supported and active.

“…When I was recovering after one of the prosthesis, despite of coming with a letter of recommendation/suggestion/threat from the orthopedic surgeon and hematologist, there was no way that…they could see me as a person who needed a treatment a little more intense…”.

(P.5 (person with hemophilia) 59 years-old)

However, they mentioned that interest in how to treat people with hemophilia is increasing.

“…there will be someone who has that know-how in terms of how to treat this type of patient”.

(P.5 (person with hemophilia) 59 years-old)

**Research**

The participants were hopeful about research, indicating that they find it to be necessary, in addition to the fact that there are already physiotherapists who know their pathology.

“…the studies that exist are very scarce and probably don’t collect practically any experience… for that I would only like to encourage some of you to work on it…”.

(P.5 (person with hemophilia) 59 years-old)

**Physical therapy as a therapy option**

Physical therapy was not previously considered as a treatment for people with hemophilia. Many participants talk about how it can help them to prevent/improve joint conditions and help them to recover from the consequences of the pathology. Parents find that counting on physical therapy has a calming effect on themselves.

“…all these talks are constructive because they teach you that… apart from medication you have another program. Especially in the case of children… It is not only medicine, but physical therapy can also help you”.

(P.6 (parent) 49 years-old)

They did not find great improvements with some tools of physical therapy. Moreover, some found a regression of the pathology due to chronification.

“In my case I would also mention the trials of myofascial therapy. The feeling was very good just after the session…After a couple of days or 3 I could feel that the effect had already worn off…”.

(P.1 (person with hemophilia) 42 years-old)

#### 3.2.2. Experience with the Conditions of Health Resources

The participants discussed their experience with the available services, in both the hospital and in the association. They also explained their experience from the past and the present. They also identified the advantages and disadvantages of their treatment.

**Health care availability**

They spoke of the predisposition and availability of health professionals.

“…there has always been predisposition by the professionals who have worked at Hospital Miguel Servet or wherever…to provide us direct information”.

(P.1 (person with hemophilia) 42 years-old)

**Adherence to the physical therapy service provided**

Some participants said that they have actually been to physical therapy, while others have not. They also highlighted whether they have been part of any research on physical therapy treatments for hemophilia.

“I have had multiple sessions with the current physiotherapist of the Association, and I have participated in some protocol of specific techniques…”.

(P.5 (person with hemophilia) 59 years-old)

“…my child…until this date, thank God, he has not needed any type of physical therapy, he did not have any injury that required it”.

(P.7 (parent) 49 years-old)

**Access to the service according to age groups: adults, teenagers, children**

They highlighted the need for educative therapy for teenagers, although it can be difficult to generate interest in them, so extra motivation must be added to attract them. They explained that they had carried out fine activities from the Association, both for children and parents.

“At the present time, you can’t do a workshop with 13-14-15-18 years-old… I don’t know how to persuade them that health education could be interesting for them”.

(P.7 (parent) 49 years-old)

**Service conditions: past and present**

They spoke about evolution in the knowledge of their pathology. They commented upon the problems derived from the pathology due to the lack of knowledge that they had previously.

“Well, older people have suffered from many things…AIDS, hepatitis…”.

(P.2 (person with hemophilia) 69 years-old)

**Organization of health resources**

They reported that it was necessary for specialist doctors to be centralized in specific treatment Units, where they know the pathology and how to deal with it.

“…that is the idea of having Hemophilia Units, in order to have specialists in all areas… but it is very complicated for us to multiply knowledge wherever we live…”.

(P.5 (person with hemophilia) 59 years-old)

#### 3.2.3. Experience with Health Education Received

They explained their experience in the educative intervention, detailing positive and negative aspects; they also talked about changes to improve future health education interventions.

**Usefulness of the briefing**

Participants found the briefing beneficial and stated that they had hoped that something new would be talked about. It generated interest in parents and older patients.

“…of course, always…anything new…that comes about our disease we are very expectative and wait for new things will come out”.

(P.2 (person with hemophilia) 69 years-old)

**Clarity of the contents**

They considered that a colloquial language has been used, so they had clearly followed the content.

“…the language was very good…you explained it so that we could understand you…in that sense it has been very good”.

(P.1 (person with hemophilia) 42 years-old)

**Strengths and limitations**

This section included the strengths and limitations of the educative intervention. The educative intervention has helped them to remember or to learn; therefore, it could be useful for parents with young children and people with arthropathies. Some of them found that it could be useful for them to apply what they have learnt in their daily lives.

“I always find that a talk has positive aspects… In a talk you will learn something, or you will be reminded about something…”.

(P.6 (parent) 49 years-old)

On the other hand, some of them found it difficult to put this into practice in their daily lives. Parents found it difficult to apply what they have learned to their children. They added that they would like to hear about techniques that are more appropriate for them.

“…I’m always with the feeling that one thing is “to watch a cooking program and another to start cooking”… Knowledge does not take up space, neither it makes feel better physically”.

(P.5 (person with hemophilia) 59 years-old)

**Health education from practice**

Participants indicated the necessity for therapeutic education from practice, and also proposed group activities where the benefits of therapeutic exercise could be seen. Another aspect where they stand out is guiding the acquired knowledge to their individual case.

“Doing group work or something like that so that people want to the benefits that you get from moving around a bit. It’s not just about being taught. It’s about being taught how to do the exercise and that’s a practical thing”.

(P.5 (person with hemophilia) 59 years-old)

**Future perspectives**

Participants highlighted the possibility of a future adapted educative intervention for younger patients. For older patients, they considered it necessary to encourage them to lead a more active life, as their condition makes it difficult for them. They added that an educative intervention based on a real case could be useful.

“Maybe for them… Focus this type of activities, talks, not selling them as talks only as a group activity… Perhaps in this indirect way, teaching them certain types of exercises as entertainment, would be beneficial for them. In this way…try to keep the body in good conditions…this would be the same for the younger age groups and the others but selling it in a different way”.

(P.5 (person with hemophilia) 59 years-old)

#### 3.2.4. A Recommended Educative Experience

They explained their previous expectations on the intervention and whether they had been achieved or not. Another aspect commented is if they would recommend this service and on which users it could be focused on.

**Health education expectations and their achievement**

They explained that the only expectation they had was to realize that someone else is worried about them and had learnt about their illness, and they were grateful for that interest. They did not give so much importance to the fact that there was not a lot of news; they were satisfied with the information received.

“What I was most looking forward to…is that someone else cares about us and there are more physiotherapists who know what they are dealing with. As soon as you know that there is a person…the expectations seem to open up a lot even though there is not much new… maybe I didn’t expect more than what we received…”.

(P.2 (person with hemophilia) 69 years-old)

**Recommend the service**

They expressed that the health education received is recommendable, although it was targeted to a very specific public. It is good that their environment knows about the disease, but they may not be interested in the briefing of hemophilia and physical therapy. They felt that it could be recommended to doctors so that they would be more informed.

“…what actually happens is that it is so specific for people with hemophilia, I don’t know who… from the association, we have been just a few participating, we are so few that all have heard about it… although it could be recommended”.

(P.2 (person with hemophilia) 69 years-old; P.7 (parent) 49 years-old)

Another participant found it difficult to believe that doctors could be interested in attend this type of interventions.

“It is very complicated because this is a rare disease…At the end, unless you go to the health center and the doctor has to see you for a long time…”.

(P.5 (person with hemophilia) 59 years-old)

### 3.3. Data Inference

Table 5 shows data inference performed after quantitative and qualitative analysis.

Each column of Table 4 expresses the different types of data, from left to right: survey dimensions, qualitative themes, survey items, qualitative subthemes, and QUAN-qual inference (expressing the relationship between both types of data). Divergence refers to the fact that the results of quantitative and qualitative analysis are opposed. Convergence means that the results of both sources of data are going in the same direction, and was found in all dimensions of the survey/themes, except for the dimension of level of loyalty.

The column “qualitative sub-themes” is related to the common significance groups that emerged after the qualitative analysis, which had a strong relationship with the different items of the quantitative survey, and expresses the idea relating both types of analysis. On the other hand, the color code refers to the different moments that participants referred to in the focus group.

## 4. Discussion

This study was conducted to examine people with hemophilia’s satisfaction with a health education presentation. The quantitative results indicate a high-level of satisfaction, although some discrepancies were found after the focus group. Four main themes came up in the data analysis:

1. Satisfaction and experience with healthcare and medical attention
2. Satisfaction and experience with health resources and conditions
3. Satisfaction and experience with health education
4. Patient loyalty and educative experience

### 4.1. Satisfaction and Experience with Healthcare and Medical Attention

Regarding the experience with healthcare and the medical attention received, high satisfaction was observed in the survey after the presentation. Despite this, there was a divergence in this result found in the focus group, where the people with hemophilia demanded more empathic health professionals with more training. As Nossair et al. indicated, health professionals should be able to communicate useful information to their patients, using all of the help available for decision making, and always having in mind the personal preferences and objectives of the people with hemophilia [29]. Halding et al. investigated how the lack of competence and flexibility in health professionals can make the collaboration difficult, and negatively influence the trust of Chronic Obstructive Pulmonary Disease (COPD) patients in them, which is why an increase in competence is needed as much as health education in self-management and rehabilitation [30]. In addition, similar results were found by Stenberg et al. in their review about how educational programs benefit the self-care of the chronic patient [31] and by Gelauff et al. in their study about education for Motor Functional Neurological Disorder [32]. These educational approaches can also improve satisfaction in some acute conditions, such as stroke patients following their experience from the hospital [33].

Regarding physical therapy, the focus group participants stated that it is a good therapeutic option, and is very necessary for prevention as well as for treatment of the long-term physical damage that their disease can have, even though there are very few studies about the effects of physical therapy in hemophilia. This idea coincides with the review by Lobet et al., which states that physical therapy has an essential role in combination with other new treatments in people with hemophilia, especially in their musculoskeletal complications [34]. In hemophilia management, the physiotherapist plays an important role in patient education and physical training, given that exercise can be helpful for them [35]. Moreover, physical therapy educational programs can reach higher patient satisfaction compared to physical therapy alone in patients at both a chronic and an acute phase [36].

### 4.2. Satisfaction and Experience with Health Resources and Conditions

In relation to their experience with health resources, the participants considered the health professionals to be available in general, but specific hemophilia units should be created. Skinner and Street remarked upon the need for a structured hemophilia unit composed of hematologists, nurses, orthopedics technicians, physiotherapists, psychologists, social workers, dentists, lab technicians and others, in order to provide specialized help and cover all of the necessities of these individuals [37]. As people with hemophilia asked during the focus group for team-based care, some studies have also found higher patient satisfaction when assigned to an interprofessional team in different chronic conditions [38,39]. Nevertheless, with regard to physical therapy, this study shows that some patients come to physical therapy while others do not. Furthermore, physical therapy is helpful for some patients, but not for others. This difference may be due to the lack of investigation and specific protocols in physical therapy for hemophilia, with some evidence pointing to the combination of physical therapy and health education as the most effective treatment [20,40].

During the focus group, different access to healthcare in different age groups was also emphasized. Health education seems to be a key approach in hemophilia, but it is very difficult to convince adolescent patients to engage, as some of their parents said. Childhood and adolescence are critical moments that can be very challenging to people with hemophilia, and constant education is needed to improve their knowledge and management of the disease, as well as to help them in their transition to adulthood [15,41].

### 4.3. Satisfaction and Experience with Health Education

In quantitative analysis, participants were generally satisfied with the information received during the online presentation in terms of benefits and clarity of the apported data. The same happened in the study by Mulders et al., who observed that an online health education program increased the knowledge and abilities of people with hemophilia [42]. However, despite the benefits of online programs, the focus group participants highlighted the possibility of health education in groups and gamified activities as an important idea for the future, especially as a way to engage younger patients. Technological innovation, telehealth and telerehabilitation, as well as videogames, could be proposed as healthcare tools set aside for the welfare of people with hemophilia, at the same time increasing their adherence to treatment and motivation [43]. Listening to these suggestions from different people in order to improve their experiences could be very interesting in terms of increasing patients’ satisfaction, as Batbaatar et al. reached to the conclusion that person-related characteristics are determinant to this outcome [44].

### 4.4. Patient Loyalty and Educative Experience

The presentation met the participants’ expectations, as they were able to comment upon different aspects of their experience with health education. In addition, there was a convergence between quantitative and qualitative analysis regarding the presentation being appealing and interesting to be recommended. The participants considered this activity and others to be useful, even if it is so specific that it can only be recommended to hemophilia sufferers and their relatives. Phadnis et al. determined the effect of a health education intervention on the parents of people with hemophilia, who increased their knowledge of the disease and its management afterwards [45]. Health education is also useful to increase the knowledge about knee replacement in hemophilia, as found by Le Dore et al., especially in children and adolescents [46]. Similar data were obtained by Breakey et al. in their study after an online self-management intervention for adolescents dealing with hemophilia, who were also prone to recommend the intervention to others [47]. Furthermore, education as an adjunct to the physiotherapy treatment can improve not only patient satisfaction, but also adherence to treatment in other chronic diseases, such as shoulder disfunction [37]. All of these findings are comparable with those of the present study, concluding the necessity for all chronic patients to have self-management information.

Another relevant outcome of this study is the need to support the participants. As Stenberg et al. inferred, health education interventions are fundamental for chronic disease patients because they not only provide knowledge, but also support and hope [31].

Regarding the existing divergences between a quantitative and qualitative analysis, they can be explained by the differences in the nature of both approaches. An evaluative survey was the chosen tool for the quantitative analysis, while not only were matters similar to the survey questions addressed during qualitative analysis, but also some other issues associated with the experience of disease. Further to this, quantitative evaluation tends to be decontextualized, whereas qualitative evaluation favors a more personalized and horizontal communication, giving researchers a wide and complete vision of the patients’ experience, even if it is not as easy to extrapolate as quantitative data [48]. Yet another factor of this divergence can be the different data extraction, given that qualitative research was performed through a focus group. This focus group leans toward a trusting and free environment that avoids social desirability bias, as concluded by Bergen et al. [49] Even with these divergences, the qualitative approach could be very useful to healthcare professionals too, as Lai et al. found in their study that asking for patients’ feedback can achieve an improved patient-centered practice [50].

## 5. Conclusions

The aim of the study was to describe the experience and satisfaction of people with hemophilia and their family members in healthcare settings. It was found that educational intervention had been positive for those patients. It should be noted that participants detected a lack of knowledge about and interest in physical therapy treatments in their disease. For this reason, physical therapy treatments and educational interventions for people with hemophilia should be further investigated.

## Figures and Tables

**Table 1:** Focus group questions.

Focus Group Questions
What was your knowledge on the topics explained before the briefing?
Have you ever had physical therapy treatments for hemophilia? Can you tell us about your experience with it?
How could you describe your experience with the briefing?
What positive/negative aspects of the briefing could you highlight?
How satisfied are you with the briefing?
Could you explain your previous expectations of the briefing?
Do you think that other people with hemophilia could benefit from the briefing? Why?
Would you recommend other people to participate in this activity?
Do you want to say anything else?

Battery of questions proposed in the focus group.

**Table 2:** Sociodemographic data of the sample (*n* = 9).

Characteristic	Data
**Age, median (SD)**	48 (13)
**Type of participants, *n* (%)**	
People with hemophilia	7 (77.8)
Family of people with hemophilia	2 (22.2)
**Gender, *n* (%)**	
Male	9 (100)
**Type of hemophilia, *n* (%)**	
Hemophilia A	6 (66.7)
Hemophilia B	3 (33.3)
**Degree hemophilia severity, *n* (%)**	
Serious	6 (75)
Moderate	1 (12.5)
Mild	1 (12.5)

SD: Standard Deviation, *n*: number of participants, %: percentage of participants.

**Table 3:** Data from GCPC-UN-ESU survey.

	1	2	3	4	5
	Lack of Satisfaction	Slightly Satisfied	Satisfied	Very Satisfied	Exceptionally Satisfied
LEVEL OF SATISFACTION WITH CARE					
1: Kindness of the staff	0%	11.1%	0%	11.1%	77.8%
2: Trust transmitted by the staff	0%	0%	0%	33.3%	66.7%
3: Preparation of the staff	0%	0%	11.1%	33.3%	55.6%
4: Interest from the staff	0%	0%	0%	11.1%	88.9%
5: Time dedicated by the staff	0%	0%	0%	11.1%	88.9%
6: Usefulness of care provided	0%	0%	11.1%	11.1%	77.8%
LEVEL OF SATISFACTION WITH THE CONDITIONS OF THE SERVICE					
7: Institutional availability	0%	0%	11.1%	22.2%	66.7%
8: Provisions for the activities	0%	0%	11.1%	22.2%	66.7%
9: Procedures to facilitate access	0%	0%	0%	22.2%	77.8%
10: Opportunity in the services	0%	0%	0%	11.1%	88.9%
11: Effectiveness in administrative conditions	0%	0%	11.1%	11.1%	77.8%
LEVEL OF SATISFACTION WITH HEALTH EDUCATION					
12: Benefits of the educational activity	0%	0%	33.3%	0%	66.7%
13: Clarity of the contents	0%	0%	0%	22.2%	77.8%
14: Appropriate educational aids	0%	0%	0%	44.4%	55.6%
15: Way of conducting the activity	0%	0%	0%	33.3%	66.7%
16: Interest raised by the topic	0%	0%	0%	33.3%	66.7%
LEVEL OF LOYALTY					
17: Fulfillment of expectations	0%	0%	0%	55.6%	44.4%
18: Would you recommend the service?	0%	0%	0%	22.2%	77.8%
19: Preference for the service	0%	0%	0%	33.3%	66.7%

Coding by levels of satisfaction: 1—Lack of satisfaction, 2—slightly satisfied, 3—satisfied, 4—very satisfied, 5—exceptionally satisfied.

**Table 4:** Themes and subthemes.

Themes	Sub-Themes
EXPERIENCE WITH HEALTH CARE	Treatment by health professionals
	Lack of trust in the health professional
	Perception of the training of professionals
	Research
	Physical therapy as a therapy option
EXPERIENCE WITH THE CONDITIONS OF HEALTH RESOURCES	Health care availability
	Adherence to the physical therapy service provided
	Access to the service according to age groups: adults, teenagers, children.
	Service conditions: past and present
	Organization of health resources
EXPERIENCE WITH HEALTH EDUCATION RECEIVED	Usefulness of the briefing
	Clarity of the contents
	Strengths and limitations
	Health education from practice
	Future perspectives
A RECOMMENDED EDUCATIVE EXPERIENCE	Health education expectations and their achievement
	Recommend the service

**Table 5:** Table of data integration (joint display).

Survey Dimensions	Qualitative Themes	Survey Items	Qualitative Subthemes	Quan-Qual Inference
LEVEL OF SATISFACTION WITH CARE	EXPERIENCE WITH HEALTHCARE	Kindness of the staff	Treatment by health professionals	DIVERGENCE
		Trust transmitted by the staff	Lack of confidence in the health professional	DIVERGENCE
		Preparation of the staff	Perception of the training of professionals	DIVERGENCE
		Interest from the staff	Research	DIVERGENCE
		Time dedicated by the staff	-----------------	----------------
		Usefulness of care provided	Physical therapy as a therapy option	DIVERGENCE
LEVEL OF SATISFACTION WITH THE CONDITIONS OF THE SERVICE	EXPERIENCE WITH THE CONDITIONS OF HEALTH RESOURCES	Institutional availability	Health care availability	CONVERGENCE
		Provisions for the activities	Adherence to the physical therapy service provided.	CONVERGENCE
		Procedures to facilitate access	Access to the service according to age group: adults, teenagers, children	CONVERGENCE
		Opportunity in the services	Service conditions: past and present	DIVERGENCE
		Effectiveness in administrative conditions	Organization of health resources	CONVERGENCE
LEVEL OF SATISFACTION WITH HEALTH EDUCATION	EXPERIENCE WITH HEALTH EDUCATION RECEIVED	Benefits of the educational activity	Usefulness of the briefing	CONVERGENCE
		Clarity of the contents	Clarity of the contents	CONVERGENCE
		Appropriate educational aids	Strengths and limitations	DIVERGENCE
		Way of conducting the activity	Health education from practice	DIVERGENCE
		Interest raised by the topic	Future perspectives	DIVERGENCE
LEVEL OF LOYALTY	A RECOMMENDED EDUCATIVE EXPERIENCE	Fulfillment of expectations	Health education expectations and their achievement.	CONVERGENCE
		Would you recommend the service?	Recommend the service	CONVERGENCE
		Preference of the service		

Color codes: 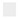 previous experience in the healthcare environment 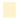 the experience with the educative intervention.

## Data Availability

Data available on request due to restrictions.
